# Human Leukocyte Antigen (HLA) and Gulf War Illness (GWI): HLA-DRB1*13:02 Spares Subcortical Atrophy in Gulf War Veterans

**DOI:** 10.1016/j.ebiom.2017.11.005

**Published:** 2017-11-09

**Authors:** Lisa M. James, Peka Christova, Brian E. Engdahl, Scott M. Lewis, Adam F. Carpenter, Apostolos P. Georgopoulos

**Affiliations:** a Brain Sciences Center, Department of Veterans Affairs Health Care System, Minneapolis, MN 5541, USA; b Department of Neuroscience, University of Minnesota Medical School, Minneapolis, MN 55455, USA; c Department of Psychiatry, University of Minnesota Medical School, Minneapolis, MN 55455, USA; d Department of Psychology, University of Minnesota, Minneapolis, MN 55455, USA; e Department of Neurology, University of Minnesota Medical School, Minneapolis, MN 55455, USA

**Keywords:** Gulf War Illness, Human Leukocyte Antigen, DRB1*13:02, DRB1*13:01, Subcortical brain atrophy, Cerebellum

## Abstract

**Background:**

Gulf War Illness (GWI) is a multisystem disorder that has affected a substantial number of veterans who served in the 1990–91 Gulf War. The brain is prominently affected, as manifested by the presence of neurological, cognitive and mood symptoms. We reported previously on the protective role of six Human Leukocyte Antigen (HLA) alleles in GWI (Georgopoulos et al., 2016) and their association with regional brain function (James et al., 2016). More recently, we reported on the presence of subcortical brain atrophy in GWI (Christova et al., 2017) and discussed its possible relation to immune mechanisms. Here we focused on one of the six HLA GWI-protective HLA alleles, DRB1*13:02, which has been found to have a protective role in a broad range of autoimmune diseases (Furukawa et al., 2017), and tested its effects on brain volumes.

**Methods:**

Seventy-six Gulf War veterans (55 with GWI and 21 healthy controls) underwent a structural Magnetic Resonance Imaging (sMRI) scan to measure the volumes of 9 subcortical brain regions to assess differences between participants with (N = 11) and without (N = 65) HLA class II allele DRB1*13:02.

**Findings:**

We found that DRB1*13:02 spared subcortical brain atrophy in Gulf War veterans; overall subcortical volume was 6.6% higher in carriers of DRB1*13:02 (P = 0.007). The strongest effect was observed in the volume of cerebellar gray matter which was 9.6% higher (P = 0.007) in carriers of DRB1*13:02 than in non-carriers. By contrast, DRB1*13:01 had no effect.

**Interpretation:**

These findings document the protective effect of DRB1*13:02 on brain atrophy in Gulf War veterans and are in keeping with recent results documenting sharing of brain mechanisms between GWI and other immune-related diseases (Georgopoulos et al., 2017). We hypothesize that the protective role of DRB1*13:02 is due to its successful elimination of external antigens to which Gulf War veterans were exposed, antigens that otherwise would persist causing low-grade inflammation and possibly leading to autoimmunity.

**Funding source:**

U.S. Department of Defense (W81XWH-15-1-0520), Department of Veterans Affairs, American Legion Brain Sciences Chair, and University of Minnesota.

## Highlights

• Subcortical brain atrophy is spared in Gulf War veterans with DRB1*13:02 allele and unaffected by the DRB1*13:01 allele.
• The strongest protective effect of the DRB1*13:02 allele was observed for cerebellar gray matter volume.
• No significant effects were observed at the DRB1*13 gene level, highlighting the importance of 4-digit HLA protein level.

Gulf War Illness (GWI) is a chronic, debilitating disorder that prominently involves the brain and is characterized, in particular, by subcortical brain atrophy. Protective effects of specific Human Leukocyte Antigen (HLA) Class II alleles on GWI have been observed, suggesting immune system involvement in GWI. Here we evaluated protective effects of DRB1*13:02 on subcortical brain volume in Gulf War veterans. The results demonstrate a protective effect of DRB1*13:02 on brain atrophy, with a marked effect on cerebellar gray matter volume. Given the goal of HLA Class II alleles is to eliminate pathogens by producing antibodies against them, the present study provides compelling insights into the likely role of persistent, pathogenic antigens resulting from the absence of DRB1*13:02 in those with GWI.

## 1 Introduction

### 1.1 Gulf War Illness

For over 25 years, veterans of the 1990–1991 Gulf War (GW) have been affected by chronic health problems, commonly referred to as Gulf War Illness (GWI), that are presumed to be sequelae of service-related exposures to toxins such as pyridostigmine bromide, pesticides, multiple vaccinations, and/or stress (White et al., 2016). Many symptoms of GWI involve the central nervous system; consequently, several studies have investigated brain structure and function as it relates to GWI, with mixed findings (White et al., 2016). We have recently identified functional (Engdahl et al., 2016) and structural (Christova et al., 2017) brain anomalies in GWI, both of which prominently involved subcortical regions. For example, compared to healthy control veterans, veterans with GWI showed an average of 10.4% reduction in cerebellar volume and 2 × the rate of reduction of cerebellar gray matter volume with age (− 14%/decade in GWI vs. − 6.9%/decade in controls). We concluded that the marked subcortical volume reduction observed in veterans with GWI is likely attributable to direct exposure to toxins, akin to toxic encephalopathy (Valk and van der Knaap, 1992), in combination with lack of immunogenetic protection in GWI (Georgopoulos et al., 2016, James et al., 2016).

### 1.2 Immunogenetics and GWI

Although a quarter to one-third of GW veterans suffer from GWI (Research Advisory Committee on Gulf War Veterans' Illnesses, 2014), most GW veterans remain relatively healthy, suggesting that genetic variations likely play a role in determining their health outcomes. In fact, we have found robust evidence that genetic variations involving the Human Leukocyte Antigen (HLA) play a substantial role in promoting protection against or vulnerability to GWI (Georgopoulos et al. 2016). HLA genes are located in the Major Histocompatibility Complex (MHC) of chromosome 6 and play a central role in immune system functioning (Meuer et al., 1982). We previously demonstrated that six HLA class II alleles (DRB1*01:01, DRB1*08:11, DRB1*13:02, DQB1*02:02, DPB*01:01, DPB1*06:01) successfully discriminate veterans with GWI from controls (Georgopoulos et al. 2016) and interact with brain function to influence symptoms of GWI (James et al., 2016). We also found an inverse relation between GWI symptom severity and the number of copies of the 6 protective HLA alleles, and that the frequency of those 6 alleles in veterans with GWI is significantly lower than in unaffected veterans (Georgopoulos et al. 2016). These effects suggest that the presence of these HLA alleles confers protection against GWI.

Notably, all 6 of the protective HLA alleles identified in relation to GWI belong to HLA class II alleles. HLA class II alleles have been strongly associated with various immune-related conditions including multiple sclerosis, rheumatoid arthritis, systemic lupus erythematosus, celiac disease, Crohn's disease, and Graves' disease, among others (Shiina et al., 2009, Gough and Simmonds, 2007). This overlap, in conjunction with several overlapping clinical signs and symptoms (Israeli, 2012), including similarities in brain synchronicity (Georgopoulos et al., 2017), places GWI squarely within the immune dysfunction realm.

### 1.3 Protective Effects of DRB1*13:02

Of the six HLA alleles previously identified as protective in terms of GWI (Georgopoulos et al. 2016), DRB1*13:02 has been found to be protective in various immune-related disorders (Bettencourt et al., 2015, Furukawa et al., 2017). Other HLA alleles have either received relatively minimal investigation in regards to their relation to autoimmune disorders, have been shown to promote susceptibility, or findings are mixed in terms of conferring susceptibility or resistance to various immune-related diseases. In a large study of associations between DRB1 alleles and six autoimmune disorders, DRB1*13 was found to be a protective factor for four autoimmune disorders (rheumatoid arthritis, systemic lupus erythematosus, psoriasis/psoriatic arthritis, and systemic sclerosis), whereas other DRB1 alleles were risk factors (Bettencourt et al., 2015). HLA DRB1*03, for instance, was strongly linked to 3 autoimmune disorders (systemic lupus erythematosus, multiple sclerosis, and myasthenia gravis). Thus, it appears that several autoimmune disorders share immunogenetic mechanisms, with DRB1*13 promoting protection, particularly for systemic and rheumatic diseases. Furthermore, the protective effects appear to be especially robust for the DRB1*13:02 allele. This protein contains 266 amino acids, of which amino acid residues at positions 30–266 form the beta chain. DRB1*13:02 contains a glycine residue at chain position 86, and differs by only one residue from the DRB1*13:01 protein which contains a valine residue at position 86. This single residue substitution makes a large difference in the electrostatic properties of pocket 9 (P9) of the peptide binding groove, i.e. the part of the HLA protein that binds to external antigens (Hov et al., 2011). DRB1*13:02 has been found to be protective against various systemic and organ-specific autoimmune disorders with gene-dosage effects conferring maximal protection in homozygous DRB1*13:02 carriers (for review, see Furukawa et al., 2017). DRB1*13:01 has also been found to protect against rheumatoid arthritis (van der Woude et al., 2010) but to be a risk factor for protracted hepatitis A infection (Pando et al., 1999) and associated pediatric autoimmune hepatitis (Fainboim et al., 2001), as well as primary sclerosing cholangitis (Hov et al., 2011). These mixed findings show that different alleles (DRB1*13:01, DRB1*13:02) can have very different disease associations, such that exploring such relations at the allele level (DRB1*13) can be misleading and uncertain. These considerations underscore the need to investigate HLA-disease associations at the protein (4-digit resolution) level, as pioneered by Todd et al. (1987) in the case of type 1 diabetes mellitus and further carried out following the publication of the crystal structures of the HLA class II molecule by Brown et al. (1993) (Jones et al., 2006).

### 1.4 The Present Study

Given the reported protective role of DRB1*13:02 for immune-related diseases and the evidence that GWI is closely related to such disorders (Georgopoulos et al., 2016, 217), we investigated the effect of DRB1*13:02 on the volumes of subcortical brain regions found to be reduced in GWI (Christova et al., 2017) to test the hypothesis that HLA DRB1*13:02 prevents subcortical brain atrophy in GW veterans, thus exerting a protective role in GWI too.

## 2 Materials and Methods

### 2.1 Participants

Seventy-six GW-era veterans (55 men, 21 women; mean age ± SEM, 53.87 ± 1.17 y) participated in the current study after providing informed consent, in adherence to the Declaration of Helsinki, and were financially compensated for their time. They included 55 veterans with GWI (52 men, 3 women) and 21 healthy controls (3 men, 18 women). All study protocols were approved by the appropriate Institutional Review Boards. GWI status was determined using a self-report symptom checklist that permits classification as GWI case or control according to the Center for Disease Control (Fukuda et al., 1998) and the Kansas criteria (Steele, 2000). All GWI veterans in the present study met both case definitions. Study participants completed diagnostic interviews including the Clinician-Administered PTSD Scale for *DSM*-*IV* (Blake et al., 1995) and the Structured Clinical Interview for DSM-IV-TR Axis I Disorders (First et al., 2002) to evaluate mental health status. None of the participants in the present study met diagnostic criteria for any mental health condition.

### 2.2 HLA Genotyping

DNA isolation was carried out from 3 ml of whole blood drawn in EDTA tubes, using a commercially available kit (ArchivePure cat. 2300730) from 5Prime (distributed by Fisher Scientific or VWR) with an expected yield of 50-150 μg of DNA. The purified DNA samples were sent to Histogenetics (http://www.histogenetics.com/) for high-resolution HLA Sequence-based Typing (SBT; details are given in https://bioinformatics.bethematchclinical.org/HLA-Resources/HLA-Typing/High-Resolution-Typing-Procedures/ and https://bioinformatics.bethematchclinical.org/WorkArea/DownloadAsset.aspx?id=6482). Their sequencing DNA templates are produced by locus- and group-specific amplifications that include exon 2 and 3 for class I (A, B, C) and exon 2 for class II (DRB1, DRB3/4/5, DQB1, and DPB1) and reported as Antigen Recognition Site (ARS) alleles as per ASHI recommendation (Cano et al., 2007).

### 2.3 MRI Data Acquisition and Preprocessing

All data were acquired using a Phillips 3T MR scanner (Achieva, Philips Healthcare, Best, The Netherlands). In the initial phase of the study, data were acquired from 42 participants using a phased array SENSitivity Encoding (SENSE) 8-channel head coil for reception. For each participant a high resolution T1-weighted Turbo Field Echo (T1w TFE SENSE) was obtained (168 sagittal slices, TR = 8.1932 ms, TE = 3.7520 ms, Acquisition matrix 240 × 240, Flip angel 8 deg., voxel size 0.9375 × 0.9375 × 1 mm). A T2-weighted image (T2w VISTA HR SENSE) was also obtained (180 slices, TR = 2500 ms, TE = 363.072 ms, Acquisition matrix 252 × 252, voxel size = 0.7813 × 0.7813 × 1 mm). Subsequently, upgrades were applied to the system and data were acquired from the remainder 34 participants using a phased array SENSitivity Encoding (SENSE) 15-channel head coil for reception. For each participant a high resolution T1-weighted Turbo Field Echo (T1w TFE SENSE) was obtained (168 sagittal slices, TR = 8.0928 ms, TE = 3.698 ms, Acquisition matrix 240 × 240, Flip angel 8 deg., voxel size 0.7500 × 0.7500 × 1 mm). The T2-weighted (T2w VISTA HR SENSE) was also obtained (168 slices, TR = 2500 ms, TE = 370.346 ms, Acquisition matrix 240 × 240, voxel size = 0.7500 × 0.7500 × 1 mm).

A 704-core High Performance Computing system (CentOS 6.5 Linux, Rocks 6.1.1) with Matlab R2012 (64 bit), Human Connectome Project (HCP humanconnectome.org) pipeline with FreeSurfer (FS; http://surfer.nmr.mgh.harvard.edu) HCP version (freesurfer-hpc) was used for data processing. MRI data with high contrast between gray matter, white matter, and cerebrospinal fluid as well as high spatial resolution are necessary for accurate results. We acquired T1w and T2w images with high spatial resolution (≤ 1 mm^2^) to achieve precise surface reconstruction. Standard FS software requires only T1w images as input. However, we used a modified version of FS, implemented in the structural HCP pipeline, which utilizes both T1w and T2w images to eliminate uncertainty due to the fact that dura and blood vessels are isointense to gray matter in the T1w image alone. In addition, T2w allows improved pial surface reconstruction (Glasser et al., 2013). Specifically, we used the first 2 structural HCP pipelines, namely *PreFreeSurfer* and *FreeSurfer*. One goal of the *PreFreesurfer* pipeline is to align the T1w and T2w images. *PreFreeSurfer* pipeline processing was followed by *FreeSurfer* pipeline processing which is based on FS version 5.2 with improvements. From the segmentation statistics output we obtained estimated total intracranial volume (eTIV), and the volumes of left and right cerebellar gray matter, brainstem, thalamus, caudate, putamen, pallidum, accumbens, amygdala and diencephalon. We calculated the sum of the left and right volumes for each region and used them as dependent variables in the ANCOVA. Finally, the sum of these subcortical volumes was the “subcortical” brain volume.

### 2.4 Data Analysis

Standard statistical methods were employed to analyze the data using the IBM-SPSS statistical package (version 23). More specifically, we carried out a univariate and a multivariate analysis of covariance (ANCOVA) to assess the effect of DRB1*13:02, DRB1*13:01 and DRB1*13 on brain volumes. In an initial analysis, we explored the possibility that the acquisition systems during the two phases of the study might have an effect on the results. For that purpose, we added a categorical “Acquisition” factor in the ANCOVAs, taking the values of 0 and 1 for the first and second phase of the study, respectively, and assessed its effect. In the univariate ANCOVA, the total subcortical volume was the dependent variable, the presence (or absence) of DRB1*13:02 was a fixed factor, and sex, age, and eTIV were covariates. Since all carriers of this HLA allele were heterozygotes in our sample, the DRB1*13:02 factor took values of zero and 1in the ANCOVA. In repeated measures ANCOVAs, the Regions (N = 9 subcortical regions) were the Within-Subjects factor (since they came from the same subject), the presence (or absence) of DRB1*13:02 was the Between-Subjects factor, and sex, age, and eTIV were covariates. The same analyses were carried out for allele DRB1*13:01.

## 3 Results

No participant carried both DRB1*13:02 and DRB1*13:01.

### 3.1 DRB1*13:02

#### 3.1.1 Frequencies

Of the total of 76 participants, DRB1*13:02 was present in 11 and absent in 65. The relative frequency of occurrence of this allele was ~ 7 × higher in controls (8/21 = 0.38) than in GWI (3/55 = 0.054), indicating a protective effect of DRB1*13:02 (Pearson chi-square = 13.08, P = 0.003; estimated odds ratio ($\hat{\omega }$) = 0.094, ln($\hat{\omega }$) = − 2.367, P = 0.001) (Table 1).

**Table 1:** Results of two-way table analysis for DRB1*13:02 and GWI.

A. Two-way table				
		Group		Total
		Control	GWI	
DRB1*13:02	Absent	13	52	65
	Present	8	3	11
Total		21	55	76

B. Analysis of the two-way table			
Test	Value	DF	Significance (2-sided)
Pearson Chi-Square	13.08	1	P = 0.0003

C. Mantel-Haenszel common odds ratio estimate			
Estimated odds ratio ($\hat{\omega }$)	ln($\hat{\omega }$)	SE of ln($\hat{\omega }$)	Asymptotic significance (2-sided)
0.094 95% lower bound: 0.022 95% upper bound: 0.403	− 2.367	0.745	P = 0.001

#### 3.1.2 Volumes

The Acquisition factor did not have any statistically significant effect in any of the DRB1*13:02-related ANCOVAs performed (P = 0.737 for Acquisition Main Effect; P = 0.805 for Acquisition X DRB1*13:02 Interaction).

**Fig. 1:**
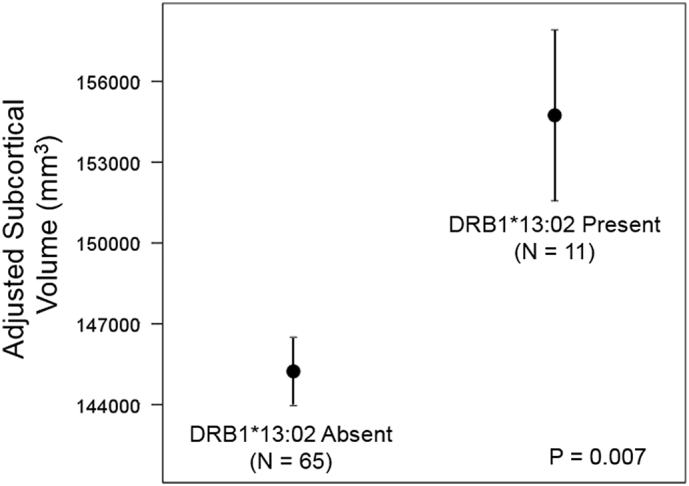
Mean (± SEM) subcortical volumes in the absence and presence of DRB1*13:02. Statistics are from a univariate ANCOVA where the Subcortical volume was the dependent variables, the absence or presence of DRB1*13:02 was a fixed factor, and sex, age and eTIV were covariates.

**Fig. 2:**
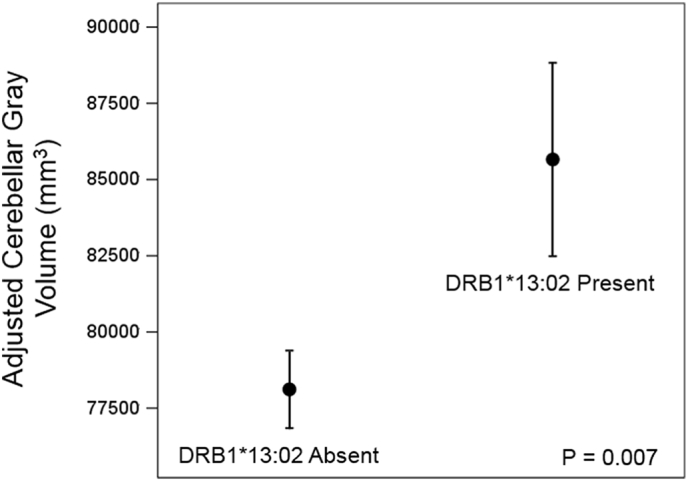
Mean (± SEM) volumes of cerebellar gray matter in the absence and presence of DRB1*13:02. Statistics are from a multivariate ANCOVA where the cerebellar gray matter volume (one of 9 subcortical regions; see Table 2) was a dependent variable, the absence or presence of DRB1*13:02 was a fixed factor, and sex, age and eTIV were covariates.

All statements on volumes below refer to volumes adjusted for sex, age and eTIV. Overall, mean volumes of the 9 subcortical regions (Table 2) were significantly higher in the presence than in the absence of DRB1*13:02 (P = 0.028, Wilcoxon Signed Rank test). In addition, the overall subcortical volume (i.e. the sum of the volumes of all 9 subcortical regions) was significantly higher by 6.6% (P = 0.007, F-test in univariate ANCOVA) (Fig. 1). A more detailed analysis was carried out using a repeated measures ANCOVA (see Methods) which revealed that the effect of the Between-Subjects DRB1*13:02 was highly significant (P = 0.007, F-test in repeated measures ANCOVA), as was the Region x DRB1*13:02 interaction (P = 0.007, Greenhouse-Geisser test), reflecting the differential effect of DRB1*13:02 on individual regions. Indeed, the strongest effect (9.6% higher in DRB1*13:02) was observed in the cerebellar gray matter (Fig. 2).

**Table 2:** Brain region volumes (mm^3^) (adjusted for sex, age, and eTIV) in the absence and presence of DRB1*13:02.

Brain region	DRB1*13:02 Absent		DRB1*13:02 Present	
	Mean	SEM	Mean	SEM
Cerebellum Gray Matter^a^	78,116.5	1000.8	85,657.3	2497.6
Brainstem^a^	21,540.1	230.7	22,537.6	575.8
Thalamus^a^	14,198.0	160.4	14,554.4	400.3
Caudate^a^	7003.6	112.5	7411.2	280.8
Putamen^a^	9839.5	139.6	9811.5	348.4
Accumbens^a^	1088.4	19.5	1122.4	48.7
Pallidum^a^	2750.9	45.9	2851.1	114.7
Amygdala^a^	3278.0	49.1	3239.9	122.6
Diencephalon^a^	7421.6	80.2	7552.5	200.1
Total Subcortical^b^	145,236.5	1270.9	154,738.0	3171.7

a Statistics from a repeated measures ANCOVA where the 9 regions were the Within-Subjects factors, the absence or presence of DRB1*13:02 was the Between-Subjects factor, and sex, age and eTIV were covariates.

b Statistics from a univariate ANCOVA where the subcortical volume was the dependent variable, the absence or presence of DRB1*13:02 was a fixed factor, and sex, age and eTIV were covariates.

### 3.2 DRB1*13:01

#### 3.2.1 Frequencies

Of the total of 76 participants, DRB1*13:01 was present in 10 and absent in 66. All DRB1*13:01 carriers belonged to the GWI group. This higher frequency of occurrence of DRB1*13:01 in GWI (18.2% vs zero) indicated an increased risk for GWI in carriers of DRB1*13:01 (Pearson chi-square = 4.397, P = 0.036; estimated odds ratio ($\hat{\omega }$) = 9.923, ln($\hat{\omega }$) = 2.29) (Table 3).

**Table 3:** Results of two-way table analysis for DRB1*13:01 and GWI. The odds ratio was estimated after adding 0.5 to all counts to avoid taking the logarithm of zero. This procedure underestimates the true effect; statistics for the odds ratio cannot be calculated.

A. Two-way table				
		Group		Total
		Control	GWI	
DRB1*13:01	Absent	21	45	66
	Present	0	10	10
Total		21	55	76

B. Analysis of the two-way table			
Test	Value	DF	Significance (2-sided)
Pearson Chi-Square	4.397	1	P = 0.036

C. Mantel-Haenszel common odds ratio estimate		
Estimated odds ratio ($\hat{\omega }$)	ln($\hat{\omega }$)	
9.923	2.295	

#### 3.2.2 Volumes

The Acquisition factor did not have any statistically significant effect in any of the DRB1*13:01-related ANCOVAs performed (P = 0.780 for Acquisition Main Effect; P = 0.975 for Acquisition X DRB1*13:01 Interaction).

Overall, mean volumes of the 9 subcortical regions (adjusted for age, sex and eTIV) did not differ significantly between carriers and non-carriers of DRB1*13:01 (P = 0.953, Wilcoxon Signed Rank test). The mean overall subcortical volume was 0.8% smaller in DRB1*13:01 carriers but not significantly different (P = 0.756, F-test in univariate ANCOVA), and similarly for the volume of cerebellar gray matter (2.0% smaller in DRB1*13:01 carriers; P = 0.592, F-test in univariate ANCOVA).

### 3.3 DRB1*13

In this analysis, the fixed factor was the allele group DRB1*13, which was deemed present when either DRB1*13:01 or DRB1*13:02 were present. No statistically significant results were yielded by any analysis.

## 4 Discussion

### 4.1 Protective Role of DRB1*13:02

In this study we investigated possible protection conferred by HLA DRB1*13:02 in GW veterans based on the facts that (a) DRB1*13:02 is protective for GWI (Georgopoulos et al., 2016), (b) DRB1*13:02 is broadly protective for immune-related disorders (Bettencourt et al., 2015, Furukawa et al., 2017, Hov et al., 2011), and (c) GWI is a neuroimmune disorder (James et al., 2016, Georgopoulos et al., 2017). Unlike typical studies based on analysis of relative frequencies of occurrence of DRB1*13:02 in various healthy and disease populations (Bettencourt et al., 2015, Furukawa et al., 2017), we, additionally, assessed its effect on subcortical brain volumes found previously to be reduced in GWI (Christova et al., 2017); indeed, we found here that DRB1*13:02 exerted a protective effect on these volumes and spared their atrophy. Specifically, the subcortical volume was significantly higher in carriers of DRB1*13:02 than in non-carriers (Fig. 1); the strongest effect was observed in the cerebellar gray matter (Fig. 2). These findings are in keeping with the overall protective role of DRB1*13:02 in immune-related disorders and in GWI, as reviewed above.

In contrast to DRB1*13:02, DRB1*13:01 had no significant effect on brain volumes in any analysis, although it was significantly more frequent in GWI. Although DRB1*13:01 has been reported to have a protective role in various immune-related diseases (Furukawa et al., 2017), it has also been reported as risk factor for autoimmune hepatitis (Duarte-Rey et al., 2009) and primary sclerosing cholangitis (Hov et al., 2011).

### 4.2 The Importance of HLA-coded Proteins

Our findings above highlight the importance of working at the HLA-protein (*β*-chain) level, which is given by the 4-digit, high-resolution HLA genotyping, as advocated by Jones et al. (2006). Most studies of HLA-disease associations in general (too many to cite), have been focused at the gene level (e.g. DRB1, DQB1, etc.) or at the allele group level (e.g. DRB1*01, DQB1*02, etc.). However, the specificity of action of a HLA allele resides on the specific HLA protein (*β*-chain) coded by it, as specified by the second set of digits in the 4-digit resolution HLA genotyping (e.g. DRB1*01:02, DPP1*06:15, etc.). Given that different HLA proteins have different properties, it follows that the proper level of analysis is at this HLA-specific protein level. Looking for HLA-disease associations at the gene or allele group levels can be misleading, yielding mixed (risk/protective) or uncertain (i.e. statistically nonsignificant) results. This problem is compounded in studies of frequencies of occurrence of various HLA alleles in different populations (e.g. healthy or suffering from a specific disease) because of the large sample sizes needed and, therefore, the increased diversity expected of HLA-specific proteins in the sample. The findings of the present study illustrate these considerations clearly because the target of the study was a concrete biological variable (i.e. volume of a brain region) and not frequency of occurrence. This afforded a clear-cut evaluation of the effect of individual HLA proteins and a contrast between the effects of either HLA protein as well as the effect of the allele group DRB1*13.

The importance of working at the HLA protein level was first demonstrated by Todd et al. (1987) in their pioneering study of the role of residue 57 of the HLA-DQ*β* polypeptide in type 1 diabetes mellitus. Recent advances in HLA protein sequencing and 3-D conformation have opened new vistas in investigating HLA-disease relations (Brown et al., 1993, Jones et al., 2006). As succinctly expressed by Donaldson, “This changed the way in which HLA associations were perceived. No longer were they seen as unexplainable genetic anomalies; it was now possible to put these associations into a functional context.” (Donaldson, 2011, p 1798). Our study rests firmly on this approach. Actually, the study by Hov et al. (2011) on the relations between HLA proteins and primary sclerosing cholangitis (PSC) is directly relevant in discussing the results of our study. Hov et al. (2011) performed a 3-D modeling of the HLA-DR*β* molecule to explore the effect of key residues on the 3-D configuration at the *β*-chain peptide binding groove. The charge of Pocket 9 (P9) of the peptide binding groove was differentially associated with PSC, such that a positive or negative charge is associated with PSC risk or protection, respectively. Specifically, Hov et al. (2011) found that in DRB1*13:01 (a risk factor for PSC; Spurkland et al., 1999) a positive P9 charge was induced by a remote action of Valine at residue 86, whereas in DRB1*13:02 (protective for PSC; Hov et al., 2011) a negative one was induced by glycine at that residue position. Extending the implications of this discovery to our study, it is reasonable to suppose that the sparing of subcortical brain atrophy we found to be associated with DRB1*13:02 is due in part to the negative charge in P9, whereas a positive charge in P9 is neutral, since DRB1*13:01 had no effect.

### 4.3 The “Persistent Antigen” Hypothesis for GWI

**Fig. 3:**
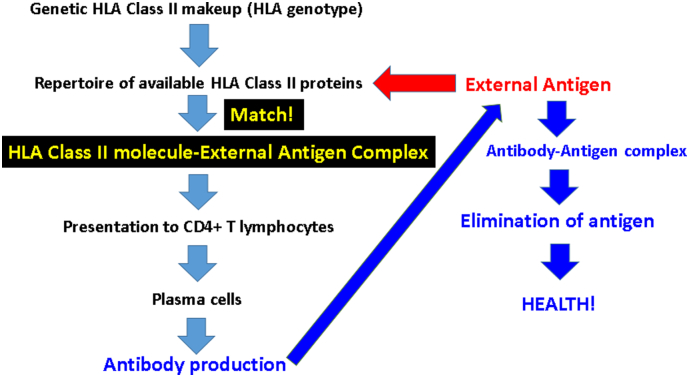
Schematic diagram illustrating the steps of antibody production in health.

**Fig. 4:**
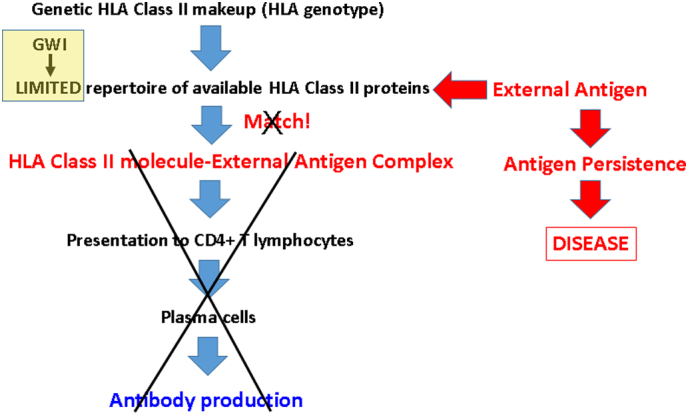
Schematic diagram illustrating the disruption at various possible stages of antibody production leading to disease.

All of the considerations above regarding the structural biological and physicochemical properties of the HLA-DR*β* peptide binding groove ultimately relate to the family of external antigens that can bind to it, to be presented to CD4 + T lymphocytes for subsequent antibody production by B cells (Fig. 3). The ultimate goal of this HLA class II-mediated specific immunity is to eliminate pathogens by producing antibodies against them. The process of successful antibody production can be disrupted at different stages, from the absence of a match between antigen and HLA class II protein (due to genetic factors) to problems with CD4 + T cells and/or plasma cell function (due to disease and/or drugs) (Fig. 4). In such cases, the external antigen/pathogen is not eliminated and can persist in the body causing inflammation and ultimately cell damage, and potentially autoimmunity through molecular mimicry (Institute of Medicine, 2012). Assuming that GW veterans were healthy when activated (in 1990–91) with respect to lymphocyte function, and given that GWI is associated with genetic lack of HLA protection (Georgopoulos et al., 2016), the most likely scenario in GWI involves a lack of antigen match with HLA class II protein, resulting in persistent, pathogenic antigen, as illustrated in Fig. 4. We call this the “Persistent Antigen Hypothesis” for GWI. Although we do not know which specific pathogens were involved in GWI, an insight can be gained from the case of pediatric autoimmune hepatitis, for which DRB1*13:01 is a risk (Fainboim et al., 2001) and DRB1*13:02 a protective factor (Pando et al., 1999). Pediatric autoimmune hepatitis frequently follows a protracted course of infection with hepatitis A virus (Fainboim et al., 2001). These authors suggested that the protracted (but not acute) hepatitis A infection leads to a sustained release of liver self-antigens, which, in turn, lead to autoimmunity (Fainboim et al., 2001). Now, DRB1*13:01 (but not DRB1*13:02) was found to be strongly associated with the protracted forms of this infection and resulting autoimmune hepatitis. Thus a connection is made between a protracted, chronic infection and a developing autoimmune disease.

### 4.4 Concluding Remarks

This line of evidence is in keeping with our “persistent antigen” hypothesis above for GWI pathogenesis. Such antigens could sustain low-grade inflammation and also lead to autoimmunity, both of which could underlie chronic inflammatory processes reported in GWI (Johnson et al., 2013). Either or both of these mechanisms (i.e. protracted low-grade inflammation and/or autoimmunity) could be involved in subcortical brain atrophy observed in GWI (Christova et al., 2017), as discussed in detail in that publication. Given the considerations above, it is possible that the protective role of DRB1*13:02 may be primarily due to preventing infection by providing “matches” (Fig. 3) for many external antigens, leading to successful production of antibodies, eliminating pathogens and thus, in the long run, preventing autoimmunity. In other words, the DRB1*13:02 protein would be a “pluripotent” HLA class II molecule. The reported protective role of DRB1*13:02 against severe malaria (Hill et al., 1991) is in keeping with this notion.

Finally, a challenge for the future is the identification of persistent antigens in GWI and their elimination. Such antigens could come from the many antigens administered to GW veterans as vaccines (Institute of Medicine National Research Council, 2000, page 295) or from other exposures, and could be at the root of the involvement of several organs systems in GWI. If identified, they could be eliminated by administering specific antibodies, e.g. as an antiserum. These possibilities are currently under investigation in our laboratory.

## Financial Disclosures

The authors do not report any financial disclosures.

## Author Contributions

Contributed to data collection and clinical evaluation: LMJ, PC, BEE, SML, AFC. Contributed to study design: APG, LMJ, PC, BEE, SML, AFC. Contributed to data analysis: LMJ, PC, APG. Wrote the paper: LMJ, APG. Contributed to editing the paper: All.

## Role of the Funding Source

Partial funding for this study was provided by the US Department of Defense, U.S. Department of Veterans Affairs, and the University of Minnesota (Brain and Genomics Fund and the American Legion Brain Sciences Chair). The sponsors had no role in the current study design, analysis or interpretation, or in the writing of this paper. The contents do not represent the views of the U.S. Department of Veterans Affairs or the United States Government.
